# Supplementary material for: Agnostic Administration of Targeted Anticancer Drugs: Looking for a Balance between Hype and Caution
Source: Int J Mol Sci. 2024 Apr 7;25(7):4094. doi: 10.3390/ijms25074094 (PMC11012409; doi:10.3390/ijms25074094)
Supplement: Supplementary file 1 [file ijms-25-04094-s001.zip › ijms-2900462-supplementary.pdf]

Supplementary Table S1. Gene names and underlying explanations.

Gene name	HGNC* approved full name	Previous name**
ABL	ABL proto-oncogene 1, non-receptor tyrosine kinase	v-abl Abelson murine leukemia viral oncogene homolog 1
AKT1	AKT serine/threonine kinase 1	v-akt murine thymoma viral oncogene homolog 1
ALK	ALK receptor tyrosine kinase	Anaplastic lymphoma kinase (Ki-1)
BCR	BCR activator of RhoGEF and GTPase	Breakpoint cluster region
BRAF	ABL proto-oncogene 1, non-receptor tyrosine kinase	v-abl Abelson murine leukemia viral oncogene homolog 1
BRCA1	BRCA1 DNA repair associated	Breast cancer 1, early onset
BRCA2	BRCA2 DNA repair associated	Breast cancer 2, early onset
CCND1	Cyclin D1	Cyclin D1 (PRAD1: parathyroid adenomatosis 1)
CHEK2	Checkpoint kinase 2	CHK2 (checkpoint, S.pombe) homolog
EGFR	Epidermal growth factor receptor	Epidermal growth factor receptor (avian erythroblastic leukemia viral (v-erb-b) oncogene homolog
FGFR1	Fibroblast growth factor receptor 1	fms-related tyrosine kinase 2
FGFR2	Fibroblast growth factor receptor 2	Bacteria-expressed kinase
FGFR3	Fibroblast growth factor receptor 3	Achondroplasia, thanatophoric dwarfism
HER2	erb-b2 receptor tyrosine kinase 2	v-erb-b2 avian erythroblastic leukemia viral oncogene homolog 2 (neuro/glioblastoma derived oncogene homolog)
KRAS	KRAS proto-oncogene, GTPase	v-Ki-ras2 Kirsten rat sarcoma 2 viral oncogene homolog
MAPK1	Mitogen-activated protein kinase 1	-
MAP2K1 (MEK)	Mitogen-activated protein kinase kinase 1	MAPK/ERK kinase 1
MET	MET proto-oncogene, receptor tyrosine kinase	met proto-oncogene
MLH1	mutL homolog 1	mutL (E. coli) homolog 1 (colon cancer, nonpolyposis type 2)
MSH2	mutS homolog 2	mutS (E. coli) homolog 2 (colon cancer, nonpolyposis type 1)
MSH6	mutS homolog 6	mutS (E. coli) homolog 6
NRAS	NRAS proto-oncogene, GTPase	neuroblastoma RAS viral (v-ras) oncogene homolog
NTRK1	Neurotrophic receptor tyrosine kinase 1	Neurotrophic tyrosine kinase, receptor, type 1
NTRK2	Neurotrophic receptor tyrosine kinase 2	Neurotrophic tyrosine kinase, receptor, type 2
NTRK3	Neurotrophic receptor tyrosine kinase 3	Neurotrophic tyrosine kinase, receptor, type 3
PALB2	Partner and localizer of BRCA2	-
PDCD1 (PD1)	Programmed cell death 1	Systemic lupus erythematosus susceptibility 2
CD274 (PD-L1)	CD274 molecule	Programmed cell death 1 ligand 1
PIK3CA	Phosphatidylinositol-4,5-bisphosphate 3-kinase catalytic subunit alpha	Phosphoinositide-3-kinase, catalytic, alpha polypeptide

PMS2	PMS1 homolog 2, mismatch repair system component	Postmeiotic segregation increased (S. cerevisiae) 2
RAD51C	RAD51 paralog C	RAD51 (S. cerevisiae) homolog C
RET	ret proto-oncogene	Multiple endocrine neoplasia and medullary thyroid carcinoma 1
ROS1	ROS proto-oncogene 1, receptor tyrosine kinase	v-ros avian UR2 sarcoma virus oncogene homolog 1
TP53	Tumor protein p53	-

* HUGO Gene Nomenclature Committee at the University of Cambridge
** Gene names often reflect rather historical context of their discovery than actual gene function or its medical significance
